# Kidney disease in very long‐term survivors of Wilms tumor: A nationwide cohort study with sibling controls

**DOI:** 10.1002/cam4.5010

**Published:** 2022-07-16

**Authors:** Stine Høgsholt, Peter Haubjerg Asdahl, Catherine Rechnitzer, Jeanette Falck Winther, Henrik Birn, Henrik Hasle

**Affiliations:** ^1^ Pediatrics and Adolescent Medicine Aarhus University Hospital Aarhus Denmark; ^2^ Department of Clinical Medicine, Faculty of Health Aarhus University Aarhus Denmark; ^3^ Department of Hematology Aarhus University Hospital Aarhus Denmark; ^4^ Pediatrics and Adolescent Medicine Copenhagen University Hospital Copenhagen Denmark; ^5^ Childhood Cancer Research Group Danish Cancer Society Research Center Copenhagen Denmark; ^6^ Department of Renal Medicine Aarhus University Hospital Aarhus Denmark; ^7^ Department of Biomedicine, Faculty of Health Aarhus University Aarhus Denmark

**Keywords:** chronic kidney disease, late effects, survivorship, Wilms tumor

## Abstract

**Background:**

Survival after Wilms tumor has significantly increased and focus on late effects has become increasingly important. However, knowledge about long‐term renal function in survivors of Wilms tumor is missing. Our aim was to investigate evidence of kidney disease in 20‐ or more‐year survivors of Wilms tumor in a clinical setting, with siblings as comparisons.

**Methods:**

In this cross‐sectional study, we established a cohort of Danish 20‐plus‐year survivors of Wilms tumor and siblings as controls. Participants answered a comprehensive health questionnaire supplemented by measurements of estimated glomerular filtration rate (eGFR), urine albumin‐to‐creatinine ratio, and blood pressure and were categorized according to the chronic kidney disease classification. Multiple linear regression analysis, taking family membership into account, was used to describe the differences in eGFR. Logistic regression analysis was performed to describe risk factors for the development of kidney disease.

**Results:**

We included 99 survivors of Wilms tumor and 38 sibling controls with a median of 37 years of follow‐up. The eGFR of Wilms tumor survivors was 13 ml/min/1.73 m^2^ (95% CI –20; −5) lower when compared to sibling control. Evidence of kidney disease, with risk factors as hypertension and diabetes, was found in 19% of the Wilms tumor survivors and 2% developed end‐stage renal disease. Ninety‐two percent of the Wilms tumor survivors had an eGFR >60 ml/min/1.73^2^.

**Conclusion:**

Long‐term Wilms tumor survivors have on average a significantly decreased renal function along with the increased prevalence of kidney disease and end‐stage renal disease when compared to sibling controls. Still, most survivors had kidney function within the normal range.

## Graphical abstract

Long‐term Wilms tumor survivors have on average a significantly decreased renal function along with increased prevalence of kidney disease and end stage renal disease when compared to sibling controls. Still, most survivors had kidney function within normal range.

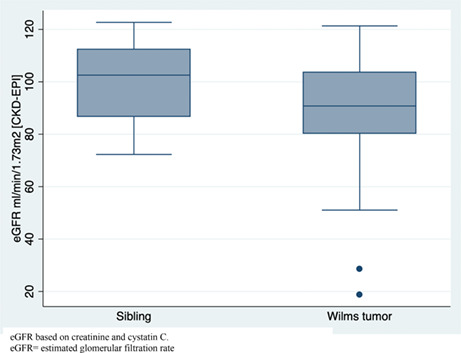

## 1 INTRODUCTION

Wilms tumor or nephroblastoma is the most common kidney cancer in children and the fifth most common childhood cancer.^1^ It affects one in every 10,000 children below the age of 15.^2^ Treatment is based on risk stratification and includes chemotherapy, surgery, and radiation therapy.^3^ Treatment is highly successful with a cure rate of almost 90%.^4^ Modern treatment for Wilms tumor is focused on minimizing the total burden of therapy, thereby reducing the risk of late effects, but maintaining the advances in cure rate.^5^, ^6^ The improvement in cure rate over the years has led to increasing numbers of long‐term survivors of Wilms tumor; however, information on the late effects following the treatment for Wilms tumor is scarce. Survivors of Wilms tumor are at increased risk of a broad range of treatment‐related late effects.^7^, ^8^, ^9^, ^10^ As the treatment for Wilms tumor includes nephrectomy often supplemented by chemotherapy and abdominal irradiation, which may affect kidney function, the risk of long‐term chronic kidney disease (CKD) is of particular concern.^11^, ^12^, ^13^ Studies have examined the risk of end‐stage renal disease (ESRD) as well as the risk of reduced glomerular filtration rate (GFR) among survivors of Wilms tumor^14^, ^15^, ^16^ identifying risk factors like nephrectomy and abdominal irradiation.^17^ Other risk factors for kidney disease and ESRD include WT1‐associated syndromes such as Denys–Drash (DDS), WAGR (Wilms tumor, aniridia, genitourinary malformations, and mental retardation/impaired cognitive function), and bilateral Wilms tumor.^15^ However, studies with sufficient follow‐up time to assess the long‐term risk and risk factors following a diagnosis of Wilms tumor are scarce.

Therefore, the aim of this study was to investigate the long‐term risk for kidney disease in 20‐plus‐year survivors of Wilms tumor.

## 2 PATIENTS AND METHODS

### 2.1 Study eligibility and recruitment

**FIGURE 1:**
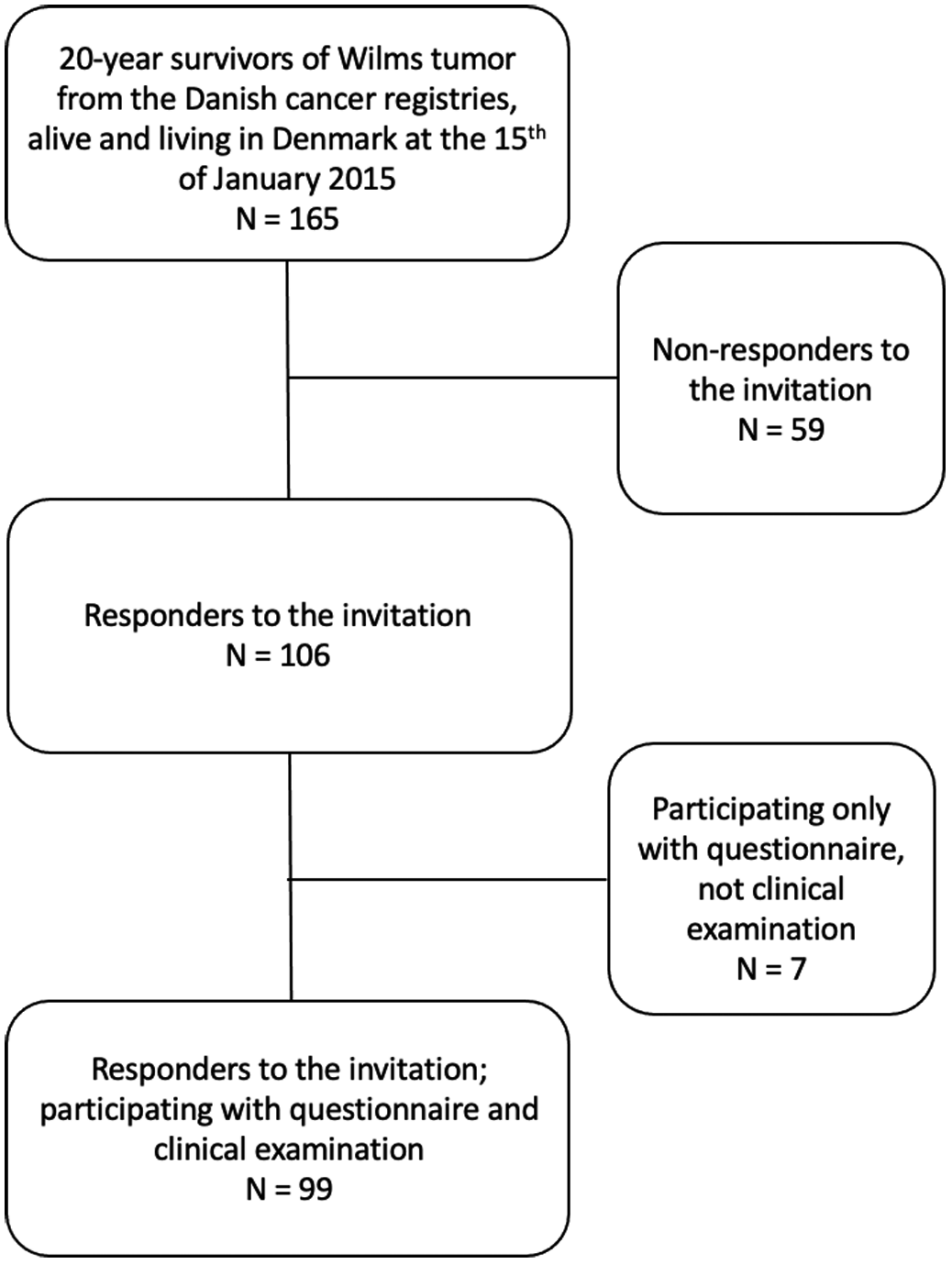
Flowchart with study eligibility, recruitment, and the final size of the Wilms tumor cohort.

In a cross‐sectional design, we established a cohort of Danish 20‐year survivors of Wilms tumor based on information from the Adult Life after Childhood Cancer in Scandinavia (ALiCCS) database.^18^ Eligibility criteria for inclusion were: (1) diagnosis of Wilms tumor reported to the Danish Cancer Registry or the Danish Childhood Cancer Registry; (2) Survival of ≥20 years from diagnosis; and, (3) alive and living in Denmark at the time point for the establishment of the cohort (January 15, 2015). Figure 1 shows the exclusion criteria. To establish a comparison cohort, siblings of the Wilms tumor survivors were invited to participate in the study.

### 2.2 Contact to the patients and their siblings

Written information explaining the purpose of the study was sent to eligible survivors of Wilms tumor. The information included a questionnaire, a consent form with the possibility to decline participation, and a prepaid return envelope. Patients not responding to the invitation received a reminder by post 4 weeks later. Those not responding within 4 weeks of the reminder were considered “non‐responders” (Figure 1). Following consent, participants were asked for permission to contact the sibling closest in age. In case of acceptance, written information similar to the one sent to the patients was sent to the siblings. The first patient was enrolled on March 24, 2015 and the last patient on August 26, 2019.

### 2.3 Comprehensive health questionnaire

The questionnaire was based on the Childhood Cancer Survivor Studies^19^ and has previously been validated in a Nordic setting.^20^ The questionnaire includes a total of 124 items assessing major domains of: (1) health status and history; (2) social and demographic factors; and, (3) health behaviors. Health status and history include current and past health problems, as well as medication use.

### 2.4 Follow‐up visit

### 2.5 Clinical examination and biochemical evaluation

All participants were invited to a clinical examination at either Copenhagen University Hospital, Odense University Hospital, or Aarhus University Hospital depending on their preference. The follow‐up included a clinical examination with blood pressure measurements, biometric measurements, urine dipstick analyses, a blood‐ and urine sampling, and an echocardiography. The clinical examinations were all performed by the same doctor (SH). The biochemical evaluation included a broad range of organ‐specific analyses. The focus of this study is on serum creatinine, cystatin C, and glycated hemoglobin A1c, along with urine albumin and creatinine. Biochemical analyses were performed at the local laboratory using standardized assays. Estimated glomerular filtration ratio (eGFR) was calculated using the combined creatinine and cystatin C equation; Chronic Kidney Disease‐Epidemiology Collaboration (CKD‐EPI).^21^, ^22^ Wilms tumor survivors and siblings were classified by the chronic kidney disease classification. Risk stratification is based on the eGFR and level of proteinuria (albumin‐to‐creatinine ratio, ACR).^21^ Study participants were registered with diabetes if they reported a diagnosis with diabetes from a doctor, active antidiabetic treatment (information from questionnaire), or had a glycated hemoglobin level > 48 mmol/mol at the follow‐up visit.

### 2.6 Blood pressure measurement

Duplicate blood pressure measurements were performed in the supine position after a minimum of 5 min rest. The mean of the two measures was used for analysis. Hypertension was defined as either systolic blood pressure > 140 mmHg, diastolic blood pressure > 90 mmHg, current treatment with antihypertensive agents, or self‐reported diagnosis with hypertension from a doctor. Information on the use of antihypertensive agents and hypertension diagnosis were self‐reported in the questionnaire.

### 2.7 Treatment abstraction

Details of treatment for Wilms tumor were retrospectively collected from medical records and abstraction of all treatment including treatment for relapse if relevant was completed. The total cumulative dose was calculated for specific chemotherapeutic agents. Cumulative epirubicin dose was converted to doxorubicin doses using a conversion factor of 0.5.^23^ Detailed information about surgery and radiation dosimetry was abstracted as well, along with information about age at diagnosis, laterality of tumor, start and end date of treatment, and information about syndromes predisposing to Wilms tumor.

### 2.8 Information on non‐responders

To assess potential bias resulting from systematic differences between responders and non‐responders, we obtained additional information on all patients with Wilms tumor available from the ALiCCS database holding information from nationwide hospital registries such as The Danish Cancer Registry, The Danish Civil Registration System, and The Danish National Patient Register (Table S1).^18^

### 2.9 Statistical analysis

Descriptive statistics were used to summarize demographic, cancer, and treatment variables for Wilms tumor survivors and sibling controls. Variables were compared using Student's *t*‐test. Multiple linear regression analysis taking family membership into account was used to compare the eGFR of Wilms tumor survivors with sibling controls. The model included sex, age at follow‐up (continuous), and irradiation (yes/no). In analyses of CKD, the outcome was treated dichotomous according to Figure 3, that is, as either normal or CKD with moderate, high, or very high risk. Logistic regression analysis was performed to describe risk factors for developing CKD. The model included sex, age at diagnosis (continuous), hypertension (yes/no), diabetes (yes/no), chemotherapy (yes/no), and irradiation (yes/no). For the sensitivity analysis where Wilms tumor survivors lost to follow‐up were compared to the study participants, follow‐up for hospitalizations started 5 years after the Wilms tumor diagnosis date. Follow‐up ended on October 31, 2010, which was the end of the ALiCCS study.^18^ If the patient had more than one hospital admission for a specific disease category, only the first admission was included in the analysis. The statistics have previously been described in detail.^8^ Data management and analyses were performed using Stata software (Stata/IC 14.2).

### 2.10 Permissions

The study's ethical approval included the possibility to contact the survivors and their siblings and to review and abstract the received treatments. The study was approved by the Danish National Committee on Health Research Ethics (1–10–72‐315‐14).

## 3 RESULTS

### 3.1 Study population and enrollment

Responses to the invitation were received from 106 of 165 (64%) eligible Wilms tumor survivors. Clinical examination was performed on 99 of the 106 survivors (Figure 1). Of the 48 siblings answering the questionnaire, 38 of them participated in the clinical examination. Thus, the total study cohort consisted of 99 Wilms tumor survivors and 38 sibling controls. Two (2%) of the Wilms tumor survivors were at ESRD, receiving chronic dialysis, and were thus excluded from the eGFR analyses.

Table 1 shows the characteristics of the study participants. Briefly, sex distribution was almost equal. The majority were diagnosed before the age of 5. The median follow‐up time was 37 years (range 23–70 years). The median age at examination was 41 years (range 24–70 years) in Wilms tumor survivors and 40 years (range 24–66 years) in the sibling control group. Three patients were treated for bilateral disease (Table 1). Additionally, two patients were diagnosed with Beckwith–Wiedemann syndrome.

**TABLE 1:** Cohort characteristics of the very long‐term survivors of Wilms tumor and sibling controls participating in the clinical examination

	Wilms tumor, *n*	Sibling comparisons, *n*
Total	99	38
Sex		
Male	49	18
Female	50	20
Tumor laterality		
Right	35	NA
Left	48	NA
Bilateral	3	NA
Missing	13	NA
Age at diagnosis		
0–4	70	NA
5–9	27	NA
10–19	2	NA
Year of diagnosis		
1947–1969	19	NA
1970–1979	34	NA
1980–1989	33	NA
1990–1994	13	NA
Years from cancer diagnosis		NA
21–30	25	NA
31–40	42	NA
41–50	23	NA
51–60	4	NA
61–70	5	NA
Age at follow‐up		
21–30	12	5
31–40	32	14
41–50	39	12
51–60	10	6
61–70	6	1
**Treatment**		
Radiotherapy		
Yes	53	NA
No	30	NA
Missing	16	NA
Radiation doses, Gray (Gy)		
10–24 Gy	8	NA
25–35 Gy	37	NA
36–45 Gy	4	NA
Missing	4	NA
Chemotherapy		
Yes	75	NA
No	8	NA
Missing	16	NA
Type of chemotherapy		
Actinomycin	75	NA
Vincristine	66	NA
Doxorubicin	19	NA
Surgery		
Yes	98	NA
No	1	NA
Missing	0	NA

Treatment information was unavailable in 16% because medical records had been destroyed or were unobtainable; however, at least 75% (90% of those with relevant information) of the Wilms tumor survivors had received chemotherapy, most of them a combination of actinomycin and vincristine, and 54% received radiotherapy. All Wilms tumors patients were treated with nephrectomy except for one with bilateral tumor (Table 1).

In total, 15% of the Wilms tumor survivors and 3% of the sibling controls were registered with diabetes and 41% of Wilms survivors and 42% of sibling controls were registered with hypertension (data not shown).

### 3.2 eGFR

**FIGURE 2:**
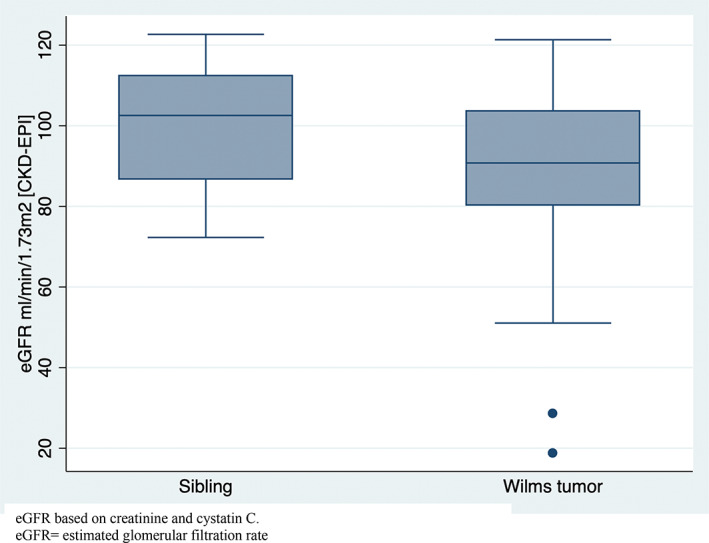
Box plot of eGFR (ml/min/1.73 m^2^) for 97 Wilms tumor survivors and 38 sibling controls.

The mean eGFR in the 97 Wilms tumor survivors was significantly lower in Wilms tumor survivors compared to the sibling control group (90 ml/min/1.73 m^2^ (95% CI 87;94) vs. 101 ml/min/1.73 m^2^ (95% CI 96;105), Figure 2). Including only the unilateral, non‐syndromic Wilms tumor survivors (*n* = 91), the results did not change this finding (data not shown). A multiple linear regression analysis taking sex, Wilms tumor diagnosis, age at follow‐up, and radiation therapy into consideration, showed that Wilms tumor survivors had eGFR that was 13 ml/min/1.73 m^2^ (95% CI –20; −5) lower than their sibling controls. A total of six (6%) (without the two patients in ESRD) Wilms tumor survivors had an eGFR <60 ml/min/1.73 m^2^, while none (0%) of the siblings had an eGFR <60 ml/min/1.73 m^2^.

**FIGURE 3:**
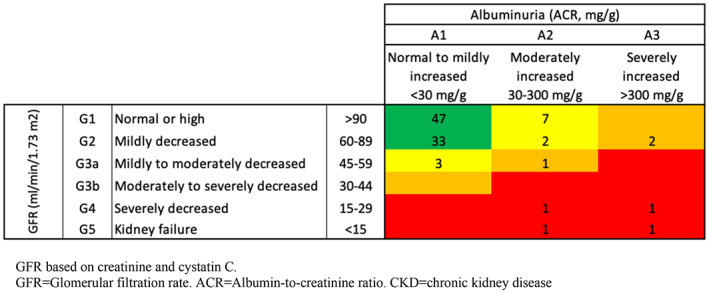
Chronic kidney disease (CKD) classification of the 99 Wilms tumor patients. Nineteen of the 99 (19%) patients are classified with CKD with moderate, high, or very high risk.

### 3.3 CKD‐classification

Albuminuria was detected in 14 (14%) Wilms tumor survivors with 4 (4%) Wilms tumor survivors revealing an ACR above 300 mg/g. In contrast, only 1 (3%) sibling had an ACR between 30 and 300 mg/g. Based on a single test, 19 (19%) Wilms tumor survivors may be defined as having CKD with moderate, high, or very high risk. The one in the sibling control group would qualify as having CKD with moderate risk. The mean age for the Wilms tumor survivors with CKD (*n* = 19) was significantly higher compared with the survivors not classified with CKD (*n* = 80) (46 vs. 40 years). The two Wilms tumor survivors on dialysis were classified as CKD stage G5/A2 and G5/A3, respectively.

### 3.4 Risk factors for CKD

Thirty‐three percent of the Wilms tumor survivors with CKD had hypertension and the odds ratio for developing CKD was 5.3 times higher when diagnosed with hypertension compared with Wilms patients without hypertension (Table 2). Similarly, a significantly increased odds ratio for CKD was seen for diabetes (OR 3.5). For age 5–9 at diagnosis and radiation therapy, the odds ratio was increased but not significant (Table 2). Notably, the two Wilms tumor survivors on dialysis were both treated with radiation therapy and diagnosed with hypertension and were 3 years of age when diagnosed. The eight patients who were not treated with chemotherapy had a low OR for CKD of 0.2 (95% CI 0.04–0.9) (Table 2).

**TABLE 2:** Risk factors for chronic kidney disease of moderate, high, or very high risk among the 99 very long‐term survivors of Wilms tumor

	*n*/number of events (%)	Adjusted odds ratio (95% CI)
Factors		
Sex		
Male	49/11 (23%)	Ref.
Female	50/8 (16%)	0.7 (0.2;1.8)
Age at diagnosia^a^		
0–4	70/13 (19%)	Ref.
5–9	27/6 (22%)	1.3 (0.4;4.0)
10–19	2/0 (0%)	NA
Hypertension^b^		
No	57/5 (9%)	Ref.
Yes	42/14 (33%)	5.3 (1.7;17)
Diabetes^b^		
No	84/13 (15%)	Ref.
Yes	15/6 (40%)	3.5 (1.1;12)
Chemotherapy		
No	8/4 (50%)	Ref.
Yes	75/12 (16%)	0.2 (0.04;0.9)
Missing	16/3 (19%)	NA
Radiation		
No	30/3 (10%)	Ref.
Yes	53/13 (25%)	2.9 (0.8;11)
Missing	16/3 (19%)	NA

^a^ Adjusted for sex.

^b^ Adjusted for sex and age at diagnosis (continuous).

### 3.5 Information on non‐responders

To assess selection bias relating to being responders, we analyzed baseline differences between responders and non‐responders (Table S1). Males were overly represented in the non‐responder group (61% vs. 49% in responder group), but with no differences in age at diagnosis. However, a bigger fraction of the non‐responders was the most recently diagnosed (1990–1994); 24% versus 12% of responders. The total number of hospitalizations due to selected diseases was 18 for responders and 12 for non‐responders. More responders (5%) have been hospitalized with chronic kidney disease than non‐responders (0%) (Table S1).

## 4 DISCUSSION

In a cross‐sectional design, with a comprehensive assessment of Danish 20‐plus‐year survivors of Wilms tumor including questionnaire and clinical examination, we evaluated markers of renal dysfunction. With a participation rate exceeding 60%, a median follow‐up of 37 years, and a total study cohort of 99 Danish 20‐plus‐year survivors of Wilms tumor and 38 sibling controls, this is the largest clinical follow‐up study in long‐term survivors of Wilms tumor.

The most obvious finding to emerge from this study is that the risk of RRT was 2% in Wilms tumor survivors compared to a lifetime risk of only 0.67% in a 20‐year‐old.^24^ Additionally, we found a significant difference in the mean eGFR between the survivors of Wilms tumor and sibling controls (90 ml/min/1.73 m^2^ (95% CI 87;94) against 101 ml/min/1.73 m^2^ (95% CI 96;105), Figure 2). The risk of CKD for survivors of Wilms tumor was highly increased compared to sibling controls; 19% of the Wilms tumor survivors had CKD with moderate, high, or very high risk, compared to only one (3%) of sibling controls, classified with CKD with moderate risk. Risk factors for developing CKD were hypertension and diabetes. Treatment with radiation therapy was associated with a non‐significant increase in OR (Table 2). This is in line with observations from a recent study, where Dieffenbach et al. found that high radiation dose to the opposite kidney and nephrectomy were independently associated with elevated risk for late‐onset kidney failure in survivors of childhood cancer, compared with sibling controls.^25^ In our study, all Wilms tumor survivors except one, were treated with nephrectomy, thus, all Wilms tumor survivors developing CKD were nephrectomized. Other studies have verified nephrectomy as an associated risk factor for decreased renal function^26^, ^27^ and nephrectomy in combination with abdominal irradiation to increase the odds of having hypertension.^26^ Dieffenbach et al. additionally concluded hypertension and diabetes to be risk factors for late‐onset kidney failure,^25^ which is in accordance with our findings (Table 2). We detected hypertension in nearly half of the Wilms tumor survivors. Even though the fraction of sibling controls with hypertension was as high, we found that hypertension was a risk factor for developing CKD in the Wilms survivors (Table 2). The recommendation to monitor blood pressure regularly in the Wilms tumor survivor group is therefore highly relevant to prevent a negative outcome on renal function.^23^ The odds ratio for developing CKD when treated with chemotherapy is below one, which most likely can be explained by the small number of patients in the reference group (*n* = 8) not treated with chemotherapy, and not that being treated with chemotherapy decreases the risk of CKD (Table 2).

In a recent study with a mean follow‐up time of 27–30 years, Green et. al demonstrated a lower eGFR in 40 survivors of Wilms tumor receiving abdominal irradiation than in controls.^13^ Green et al. also used, among others, the combined CKD‐EPI estimating equation with serum creatinine and cystatin C. In their study, no survivors had an eGFR <60 ml/min/1.73^2^ using the combined CKD‐EPI estimating equation, and one survivor (5%) was classified with CKD, characterized by decreased eGFR with or without proteinuria.^13^ The differences from our study, where 8% of the Wilms tumor survivors had an eGFR <60 ml/min/1.73^2^, and 19% were classified with CKD might be due to the differences in cohorts regarding risk factors for decreased GFR, such as age, ethnicity, comorbidity, or simply due to the differences in sample size. We also evaluated renal function as the estimated glomerular function renal calculated from the combined creatinine–cystatin C equation (CKD‐EPI) taking age, sex, and race into consideration. This equation using the two plasma markers is proven to provide the most precise and accurate estimate of GFR compared with equations based on either creatinine or cystatin C alone.^22^

There were no patients in our cohort with Denys–Drash or WAGR syndrome. These patients have been reported to have a higher incidence of ESRD than unilateral, non‐syndromic Wilms tumor survivors.^15^, ^16^, ^17^, ^18^, ^19^, ^20^, ^21^, ^22^, ^23^, ^24^, ^25^, ^26^, ^27^, ^28^ Two patients in our cohort were diagnosed with Beckwith–Wiedemann syndrome, the most common overgrowth and cancer predisposition syndrome.^29^ Three patients in our cohort were treated for bilateral tumor. Bilateral Wilms tumor is a risk factor for ESRD.^15^, ^16^, ^17^, ^18^, ^19^, ^20^, ^21^, ^22^, ^23^, ^24^, ^25^, ^26^, ^27^, ^28^ A sensitivity analysis showed the same difference in eGFR after excluding the five patients with risk factors. Therefore, we decided to keep the five patients in the cohort for the following analyses.

The results of this study should be evaluated in the light of certain limitations. Firstly, our study is a cross‐sectional study based on a single follow‐up visit. Due to this study design, we do not have repeated measurements and our data do not enable us to describe the development of renal function over time. We can only conclude from the one‐time measurements, and we do not have the information on whether or not any renal outcome is developed earlier in life, for example, in relation to the treatment for Wilms tumor and persisted, or if it has developed over time. Additionally, we cannot conclude whether or not the renal outcome is chronic, as this would require two separate examinations with a minimum of 3 months between.^21^ For the age comparison between survivors classified with CKD to the ones without, showing that the survivors classified with CKD were significantly older, one could speculate if the younger participants may develop renal dysfunction later in life. This emphasizes the rationale to maintain surveillance of the renal function in the Wilms tumor survivors. It might also be the result of more intensive treatment protocols, that is, more use of radiation therapy in the older patients. In addition, we cannot conclude on the cause‐and‐effect relationship, for example, if diabetes or hypertension is developed before the decrease in renal function. Secondly, we do not have information on mortality or cause of death. A study about early and late mortality in Wilms tumor survivors reported 8% mortality from 5 to 20 years after diagnosis.^30^ Applying this to our cohort, a total of 14 patients may have died in this period, but as we do not have the information on cause of death, we cannot rule out if some of the patients could have died due to severe renal late effects, and that we, therefore, underestimate the prevalence of ESRD. Furthermore, it is important to notice that even though the number of long‐term survivors of Wilms tumor participating in this study is high, the number of sibling controls is smaller at 38. A major limitation in studies examining the health condition of survivors by clinical examination is the bias that may arise due to systematic differences between responders and non‐responders. In our study, 36% of the invited participants did not respond to the invitation, which could cause selection bias. Therefore, we included a description of differences in characteristics and morbidity between responders and non‐responders (Table S1). Non‐responders and responders were similar regarding demographic and cancer‐related characteristics, but with the majority of males among non‐responders. The hospitalization diagnoses within the different organ systems; circulatory, urinary, digestive, endocrine, and bone were selected to reflect the specific investigations completed on responders at the follow‐up visit. The number of hospitalizations was overall comparable, which minimizes the risk of selection bias in this study. The number of responders hospitalized with chronic renal failure was higher than the non‐responders. Thus, it is unlikely that we underestimate the incidence of kidney disease in the study cohort. The fraction of non‐responders in the most recent years of diagnosis (1990–1994) was higher. Because we hypothesize that the largest risk of renal late effects is seen among survivors diagnosed in the earlier years, due to more treatment overall, specifically more radiation therapy,^31^ we likely do not underestimate the risk of renal late effects in this study.

To conclude, we found that a fraction of the 20‐plus‐year survivors of Wilms tumor has a significantly increased risk for ESRD, decreased renal function, increased prevalence of albuminuria, and CKD with moderate, high, or very high risk in comparison to their non‐cancer sibling controls. The decrease in eGFR among survivors of Wilms tumor is likely to be minor, and importantly 92% of the Wilms tumor survivors had an eGFR >60 ml/min/1.73.^2^ A significant fraction had albuminuria, and 2% of the Wilms tumor survivors developed ESRD and were in need of treatment with dialysis. The results of this study underline that most patients will have a good lifelong kidney health despite tumor location and treatment with nephrotoxic potential. This may be comforting for the parents and health‐care professionals of newly diagnosed Wilms tumor patients regarding risks of kidney disease. However, our study also emphasizes the importance of following Wilms tumor survivors and identifies modifiable risk factors as hypertension and diabetes in the advantage of the risk of developing chronic kidney disease.

## AUTHOR’S CONTRIBUTION

Stine Høgsholt: conceptualization, funding acquisition, investigation, methodology, project administration, resources, validation, visualization, writing ‐ original draft.

Peter Haubjerg Asdahl: supervision, methodology, validation, visualization, writing review, and editing.

Catherine Rechnitzer: supervision, validation, visualization, writing review, and editing.

Jeanette Falck Winther: supervision, validation, visualization, writing review, and editing.

Henrik Birn: conceptualization, methodology, supervision, validation, visualization, writing review, and editing.

Henrik Hasle: conceptualization, methodology, supervision, resources, validation, visualization, writing review, and editing.

## FUNDING INFORMATION

This study was supported by grants from the Danish Cancer Society (Grant number: R150‐A10178‐16‐S48) and the Danish Childhood Cancer Foundation (Grant number: 2014–33). The funding sources played no role in the design or analysis of the study nor in the interpretation of its findings. No conflicts of interest were declared.

## CONFLICT OF INTEREST

None of the authors have conflict of interests to declare.

## Supporting information


Table S1

## Data Availability

Due to privacy and ethical restrictions, data cannot be shared
