# Supplementary material for: Kidney disease in very long‐term survivors of Wilms tumor: A nationwide cohort study with sibling controls
Source: Cancer Med. 2022 Jul 16;12(2):1330–8. doi: 10.1002/cam4.5010 (PMC9883410; doi:10.1002/cam4.5010)
Supplement: Supplementary file 1 — Table S1 [file CAM4-12-1330-s001.docx]

| **Supplementary Table 1** Differences in characteristics between responders (n=97) and non-responders (n=62) | | |
| --- | --- | --- |
|  | Responders^a^ | Non-responders^a^ |
| Total | 97 | 62 |
| **Sex, n** |  |  |
| Male | 49 | 38 |
| Female | 48 | 24 |
| **Age at diagnosis, n** |  |  |
| 0-4 | 70 | 42 |
| 5-9 | <30 | <20 |
| 10-19 | <5 | <5 |
| **Year of diagnosis, n** |  |  |
| 1947-1969 | 19 | 9 |
| 1970-1979 | 32 | 17 |
| 1980-1989 | 34 | 21 |
| 1990-1994 | 12 | 15 |
| **Years from cancer diagnosis** |  |  |
| 21-30 | 30 | 29 |
| 31-40 | 38 | 17 |
| 41-50 | 21 | 10 |
| 51-60 | 4 | <5 |
| 61-70 | 4 | <5 |
| **Hospitalizations, n** |  |  |
| Total number | 18 | 12 |
| **Circulatory system** |  |  |
| Heart failure | <3 | <3 |
| Congestive heart failure | 0 | <3 |
| Cardiomyopathy | <3 | <3 |
| **Malignant neoplasms (new primary cancer)** |  |  |
| Cancer of endocrine organs | <3 | <3 |
| **Urinary system** |  |  |
| Chronic kidney disease | 5 | 0 |
| Infections of the urinary system | <3 | <3 |
| **Digestive organs** |  |  |
| Acute pancreatitis | 0 | <3 |
| Other chronic pancreatitis | <3 | <3 |
| **Endocrine diseases** |  |  |
| Insulin-dependent diabetes mellitus | <3 | <3 |
| Insulin independent diabetes mellitus | <3 | <3 |
| **Diseases of bone, joint and soft tissue** |  |  |
| Scoliosis | <3 | <3 |
| ^a^The table only includes patients registered in the Danish Cancer Registry. Two of the responders and four of the non-responders were not registered in the Danish Cancer registry but in the Danish Childhood Cancer Registry, which is why numbers are 97 and 62 for responders and non-responders, respectively.  According to EU’s General Data Protection Regulation (GDPR), it is important to avoid that any individual can be identified. That is why we for certain rare diseases do not provide the exact number of observations, but use the “less than or equal to” observations (≤) | | |
